# Comparative Evaluation of the Effect of Radiation on the Marginal Adaptation of Different Composite Restorative Materials: An In Vitro Study

**DOI:** 10.7759/cureus.113805

**Published:** 2026-08-02

**Authors:** Sakshi Singla, Manu Bansal, Rajinder Bansal, Tapas K Dora, Mamta Singla, Manmeet Kaur, Supriya Chaudhary

**Affiliations:** 1 Department of Conservative Dentistry and Endodontics, Guru Nanak Dev Dental College and Research Institute, Sunam, IND; 2 Department of Radiation Oncology, Homi Bhabha Cancer Hospital, Sangrur, IND; 3 Department of Conservative Dentistry and Endodontics, Faculty of Dental Sciences, Shree Guru Gobind Singh Tricentenary University, Gurugram, IND

**Keywords:** composite resins, dental restoration, marginal adaptation, permanent, radiotherapy, scanning electron microscopy

## Abstract

Background: Radiotherapy is an essential component of the management of head and neck cancers; however, it adversely affects both dental hard tissues and restorative materials, potentially compromising the marginal integrity of restorations. As marginal adaptation is a key determinant of restoration longevity, evaluating the performance of different resin-based restorative materials under irradiated conditions is of clinical importance. The aim of this study was to comparatively evaluate the effect of therapeutic radiation on the marginal gap of different composite restorative materials using an in vitro model.

Materials and methods: A total of 36 freshly extracted human premolars were randomly assigned to four groups (n = 9 each) according to the restorative material used: nanofilled composite resin, giomer, ormocer, and bulk-fill flowable composite (SureFil SDR Flow Plus, Dentsply Sirona, Charlotte, NC). Standard class V cavities were prepared and restored according to the manufacturers' recommendations. The marginal gap at the tooth and filling material junction was assessed using scanning electron microscopy before and after exposure to a cumulative radiation dose of 70 Gy, delivered according to the conventional head and neck radiotherapy protocol. Statistical analysis was performed using one-way analysis of variance (ANOVA), Tukey's honestly significant difference post-hoc test, paired t-test, and Shapiro-Wilk test. Statistical significance was set at p < 0.05.

Results: Before radiation exposure, a statistically significant difference in the marginal gap was observed between the four restorative materials (p < 0.001). Ormocer exhibited the lowest mean marginal gap (4.27 ± 0.32 µm), whereas giomer demonstrated the highest (6.30 ± 0.42 µm). After radiation exposure, the significant intergroup differences persisted (p < 0.001). Ormocer continued to show the lowest marginal gap (4.86 ± 0.30 µm), while SureFil SDR Flow Plus exhibited the highest (7.08 ± 0.30 µm). Intragroup analysis revealed a significant increase in the marginal gap following radiation exposure for all restorative materials (p < 0.05), with the greatest effect observed in the SureFil SDR Flow Plus group (Cohen's d = 2.38).

Conclusion: Therapeutic radiation significantly increased the marginal gap of all evaluated restorative materials. Among the materials tested, ormocer demonstrated the best marginal adaptation and the greatest resistance to radiation-induced deterioration, suggesting its potential clinical advantage in restoring teeth in patients undergoing head and neck radiotherapy.

## Introduction

Head and neck cancers are among the most common malignancies worldwide, and radiotherapy remains one of the primary treatment modalities for their management [1]. Although radiotherapy improves patient survival, ionizing radiation adversely affects the oral environment by causing salivary gland dysfunction, xerostomia, reduced buffering capacity, altered tooth structure, and increased susceptibility to radiation-induced dental caries [2]. These changes often necessitate the placement or replacement of direct resin-based restorations, either before or after the completion of radiotherapy. Therefore, the long-term clinical success of restorative materials in irradiated teeth has become an important concern in restorative dentistry [3].

One of the key determinants of restoration longevity is marginal adaptation. An inadequate marginal seal permits bacterial infiltration, microleakage, postoperative sensitivity, marginal discoloration, recurrent caries, and ultimately restoration failure [4]. Exposure to therapeutic radiation may alter the physical and mechanical properties of both dental hard tissues and restorative materials by affecting the polymer integrity, filler-matrix interaction, and adhesive bonding at the tooth-restoration interface. Consequently, restoration margins may become more susceptible to degradation after radiation therapy [5,6].

Recent advances in restorative dentistry have introduced several resin-based materials with different compositions and filler technologies, including nanofilled, giomers, ormocer-based, and bulk-fill flowable composites. These materials differ in their polymer matrix, filler characteristics, polymerization behavior, and fluoride-releasing capabilities, which may influence their ability to maintain marginal integrity under irradiation [7,8]. Despite their widespread clinical use, limited evidence is available to compare marginal adaptation before and after exposure to therapeutic radiation. Identifying restorative materials that demonstrate superior marginal stability following irradiation would help clinicians make evidence-based decisions when restoring teeth in patients undergoing radiotherapy for head and neck cancers.

The aim of the present study was to comparatively evaluate the effect of therapeutic radiation on the marginal adaptation of four commonly used resin-based restorative materials: nanofilled composite, giomer, ormocer, and bulk-fill flowable composite using an in vitro model. The objectives of this study were to assess and compare the marginal gap exhibited by these restorative materials before and after radiation exposure, determine the influence of radiation on their marginal integrity, and identify the material that demonstrates the most favorable marginal adaptation under irradiated conditions.

## Materials and methods

The present in vitro experimental study was conducted between May 2024 and December 2024 at the Department of Conservative Dentistry and Endodontics, Guru Nanak Dev Dental College and Research Institute, Sunam, Punjab, India, to evaluate the effect of therapeutic radiation on the marginal adaptation of different resin-based restorative materials. Prior to the commencement of the study, approval was obtained from the Institutional Ethics Committee (Ref No: GNDDC/2024/348) for the use of extracted human teeth.

The sample size was estimated using G*Power software version 3.1.9 (Heinrich Heine University Düsseldorf, Düsseldorf, Germany) by performing an a priori power analysis under the F-test family. Based on an effect size of 0.58, derived from a previously published study evaluating marginal gaps among composite restorative materials, with the statistical power set at 80% and the level of significance (α) fixed at 5%, the minimum required sample size was calculated as 36 specimens [9]. The specimens were equally allocated to four experimental groups, each consisting of nine teeth, to ensure adequate statistical power for intergroup comparisons.

A total of 36 freshly extracted human premolars extracted for orthodontic purposes were collected from the Department of Oral and Maxillofacial Surgery of the same institution. Immediately after extraction, all specimens were cleaned thoroughly using an ultrasonic scaler to remove adherent soft tissue remnants and calculus. The teeth were then disinfected by immersion in a 5% sodium hypochlorite solution for 24 hours and subsequently stored in 0.9% normal saline until further use to prevent dehydration. Only intact permanent premolars extracted for orthodontic or periodontal reasons were selected based on eligibility criteria (Table 1).

**Table 1:** Inclusion and exclusion criteria for tooth selection.

Inclusion criteria	Exclusion criteria
Intact permanent premolars	Dental caries
Sound tooth structure (no defects)	Restorations (any type)
Clinically intact buccal and occlusal surfaces	Developmental anomalies
No evidence of pathological wear	Cervical abrasions or erosion
No previous endodontic intervention	Cracks or fractures

The 36 selected teeth were randomly allocated into four experimental groups (n = 9 per group) using a computer-generated randomization sequence. Each specimen was assigned a unique identification number from 1 to 36, and the allocation sequence was generated using the random-number function in Microsoft Excel (Microsoft Corporation, Redmond, WA). The generated sequence was used to assign the specimens in a 1:1:1:1 ratio to the four experimental groups. Group I specimens were restored with a nanofilled composite resin (Filtek™ Z350 XT, 3M ESPE, St. Paul, MN), Group II with a giomer restorative material (Beautifil II™, Shofu Inc., Kyoto, Japan), Group III with an ormocer-based composite (Admira Fusion®, VOCO GmbH, Cuxhaven, Germany), and Group IV with a bulk-fill flowable composite (SureFil™ SDR™ Flow Plus, Dentsply Sirona, Charlotte, NC). Each specimen served as its own control, and marginal adaptation was evaluated before and after radiation exposure.

Standardized class V cavities measuring 3 mm mesiodistally × 3 mm occlusogingivally × 2 mm in depth were prepared on the buccal surfaces of all teeth using a high-speed air turbine handpiece (Apple Dental, Mumbai, India) fitted with a cylindrical diamond bur (Mani Inc., Tochigi, Japan) under a continuous water coolant. The occlusal margin of each cavity is entirely located in the enamel, whereas the cervical margin extends into the dentin. To maintain a uniform cutting efficiency, a new bur was used after every five cavity preparations. The cavity dimensions were verified using a Williams graduated periodontal probe (API, New Delhi, India).

Following cavity preparation, selective enamel etching was performed using a 37% phosphoric acid etchant (Neoetch Gel, Neoendo, New Delhi, India) for 15 seconds. The cavities were rinsed thoroughly with water for 10 seconds and gently air-dried while maintaining the moist dentin. A universal adhesive (Single Bond Universal Adhesive, 3M ESPE, St. Paul, MN) was applied according to the manufacturer's instructions, gently air-thinned for five seconds, and light-cured for 20 seconds using a light-emitting diode (LED) light-curing unit (RTA Minis, Guilin Woodpecker Medical Instrument Co., Ltd., Guangxi, China) with an output intensity of approximately 1000 mW/cm².

Each experimental group was restored using its designated restorative material according to the manufacturer's instructions. Composite placement was performed carefully to ensure complete adaptation to the cavity walls, followed by light curing for 20 seconds. The restorations were subsequently finished and polished using a Sof-Lex™ Finishing and Polishing System (3M ESPE, St. Paul, MN). Immediately after extraction and disinfection, the teeth were stored in 0.9% normal saline at room temperature until further use to prevent dehydration. Throughout the experimental period, whenever the specimens were not undergoing cavity preparation, restorative procedures, scanning electron microscope (SEM) evaluation, or irradiation, they were maintained completely immersed in fresh 0.9% normal saline. The storage solution was renewed every 24 hours to maintain consistent storage conditions and minimize changes in the storage medium. Following restoration, the specimens were similarly stored in fresh 0.9% normal saline for 24 hours before baseline evaluation. During the seven-week irradiation period, the specimens were returned to fresh normal saline immediately after each irradiation session and remained immersed until the subsequent fraction.

**Figure 1:**
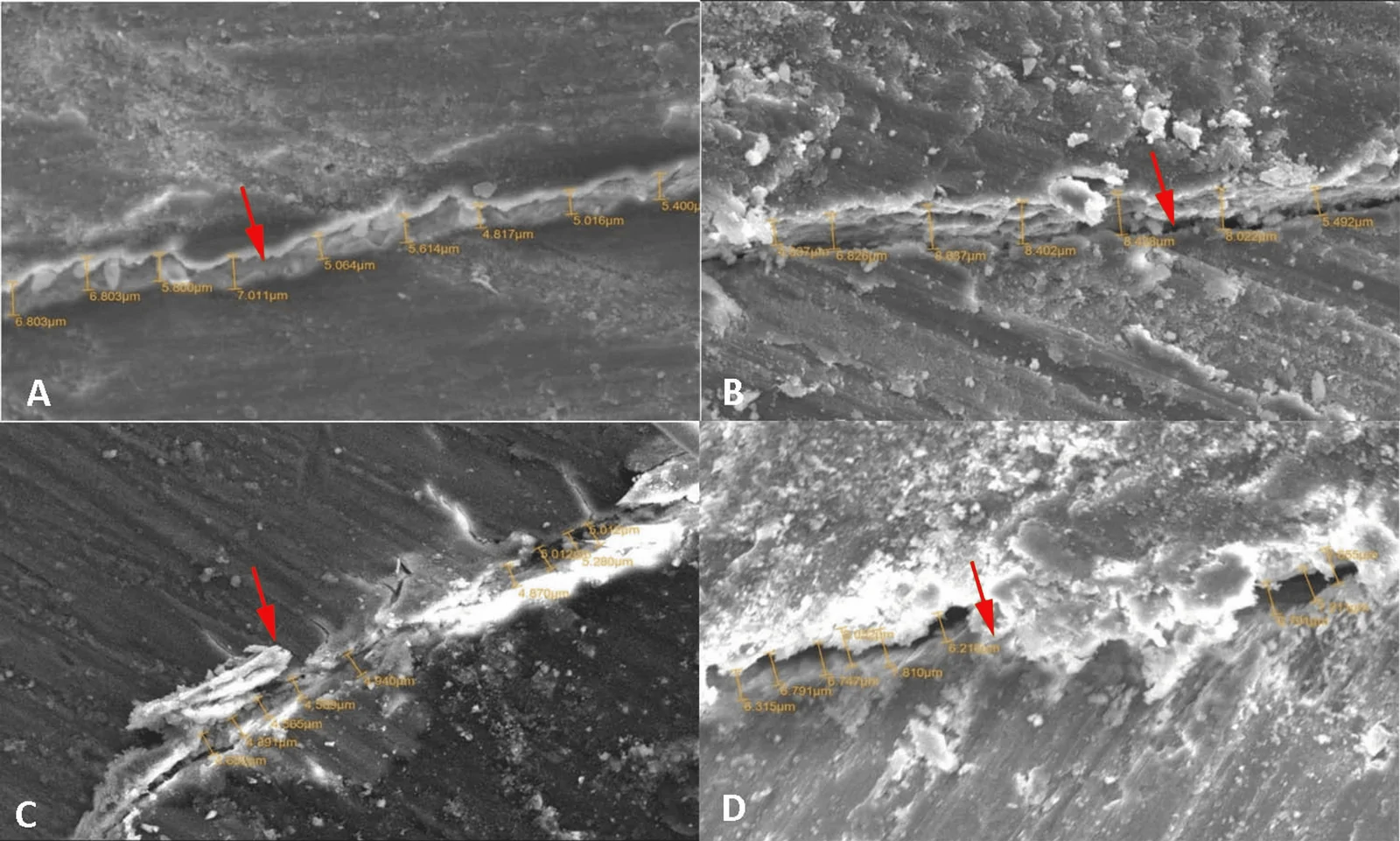
Scanning electron microscopy (SEM) showing a marginal gap (red arrows) in each group specimen before radiation exposure: (A) Group I, (B) Group II, (C) Group III, and (D) Group IV. SEM image magnification at 1000x. Three measurements were taken from the specified location and averaged.

Baseline marginal adaptation was evaluated by sectioning each restored tooth longitudinally through the center of the restoration in a buccolingual direction, using a diamond-coated disc (R & D Impex International, Ludhiana, India) under continuous water irrigation. The sectioned specimens were dehydrated and sputter-coated with gold before examination under a scanning electron microscope (JSM-6510LV; JEOL Ltd., Tokyo, Japan). Standardized photomicrographs were obtained under identical magnification for all specimens, and the marginal gap at the tooth-restoration interface was measured in micrometers (µm). Three measurements were recorded for each specimen at predetermined locations, and the average values were used for statistical analysis (Figure 1).

Following baseline evaluation, all specimens were subjected to therapeutic irradiation using an Elekta Versa HD Linear Accelerator (Elekta AB, Stockholm, Sweden). The specimens were positioned in a reproducible manner within a custom-made water-equivalent phantom, with the restored buccal surfaces oriented perpendicular to and facing the incident radiation beam. The specimens were arranged centrally within the irradiation field and completely surrounded by water-equivalent material to provide adequate lateral scatter and simulate tissue-equivalent conditions. Irradiation was performed using a 6-MV photon beam with a field size of 10 × 10 cm² at a source-to-surface distance (SSD) of 100 cm. The specimens were positioned at a depth of approximately 5 cm within the water-equivalent phantom. A 1.5-cm-thick tissue-equivalent bolus was placed over the specimen region to provide adequate dose build-up and ensure that the tooth restoration interfaces were encompassed within a region of uniform dose deposition.

**Figure 2:**
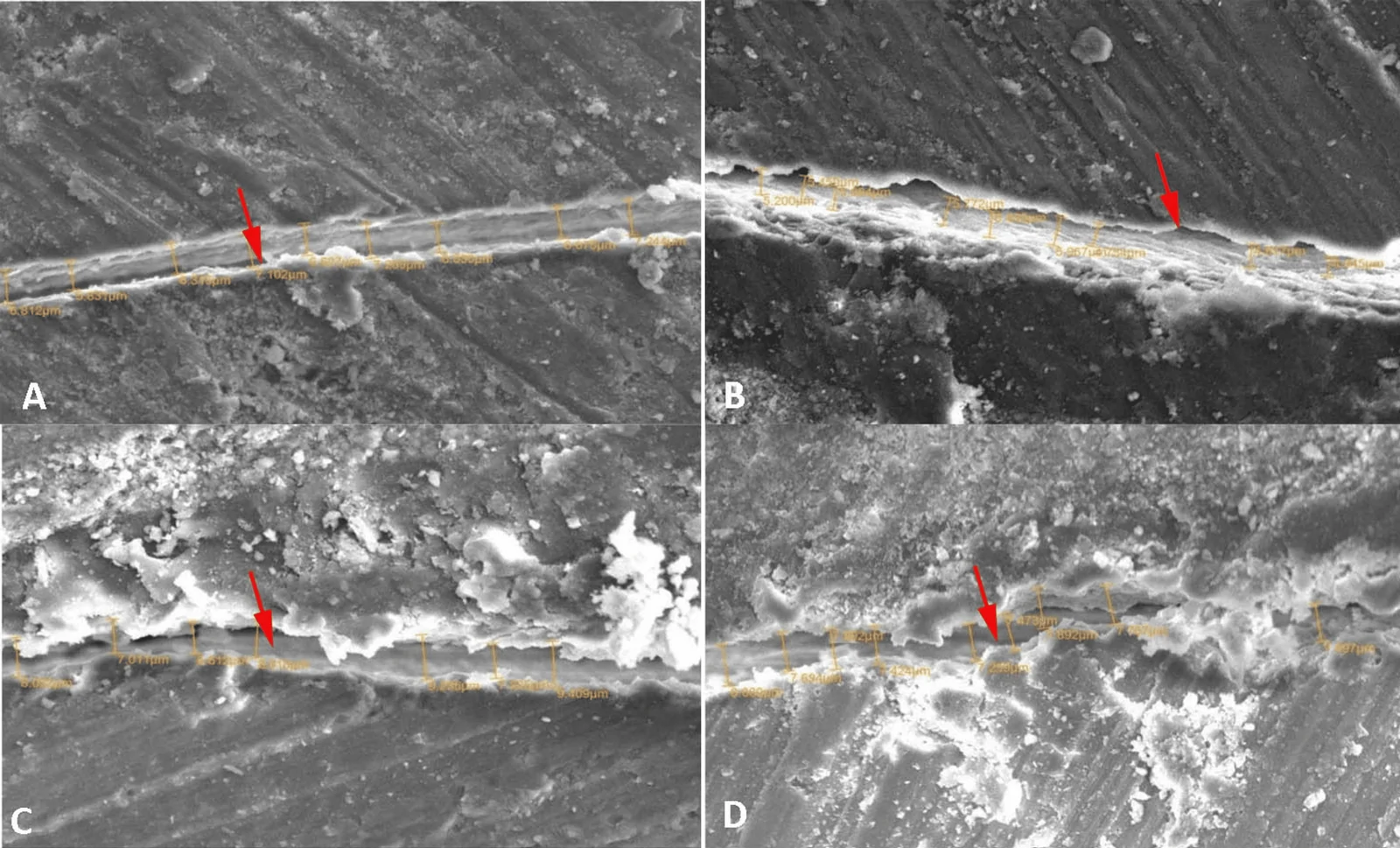
Scanning electron microscopy (SEM) showing a marginal gap (red arrows) in each group specimen after radiation exposure: (A) Group I, (B) Group II, (C) Group III, and (D) Group IV. SEM image magnification at 1000x. Three measurements were taken from the specified location and averaged.

A cumulative radiation dose of 70 Gy was delivered in 35 fractions of 2 Gy each, administered once daily, five days per week, for seven consecutive weeks, corresponding to a conventional fractionation schedule used for head and neck radiotherapy [10]. The irradiation field was maintained consistently throughout all treatment sessions. Dose delivery and uniformity at the specimen level were verified by the medical physicist using the linear accelerator dosimetry system and treatment-planning calculations, with the prescribed dose normalized to the specimen plane. After completion of the radiation protocol, marginal adaptation was reassessed using the same SEM under identical imaging conditions and magnifications employed during the baseline evaluation. Marginal gap measurements were obtained for each group specimen (Figure 2) using the same standardized protocol, and the average of three measurements per specimen was used for the final statistical analysis.

For each specimen, marginal gap measurements were obtained at three standardized predetermined locations along the tooth-restoration interface: (1) the occlusal margin, corresponding to the enamel-restoration interface; (2) the midpoint of the cavity wall, defined as the point equidistant between the occlusal and cervical margins; and (3) the cervical margin, corresponding to the dentin-restoration interface. At each location, the perpendicular distance between the restorative material and the adjacent cavity wall was measured in micrometers (µm) on the SEM photomicrograph. The three measurements were recorded under identical magnification and imaging conditions, and their arithmetic mean was considered the representative marginal-gap value for that specimen for statistical analysis. The same standardized measurement locations were used for both the pre-radiation and post-radiation evaluations.

To minimize operator-related variability, all cavity preparations, adhesive procedures, restorative material placement, light curing, finishing, and polishing were performed by a single trained and calibrated operator. Prior to commencement of the study, examiner calibration was performed to ensure measurement reliability. The principal investigator underwent training under the supervision of an experienced faculty member for the SEM evaluation of marginal adaptation. Ten additional extracted teeth that were not included in the study sample were independently evaluated twice by the principal investigator with a two-week interval between assessments.

Reliability was assessed using a two-way random-effects, absolute-agreement, single-measure intraclass correlation coefficient (ICC). Excellent measurement reproducibility was demonstrated, with an intra-examiner ICC of 0.94 (95% CI: 0.81-0.98) and an inter-examiner ICC of 0.92 (95% CI: 0.73-0.98). An ICC value ≥0.90 was considered indicative of excellent reliability. These findings confirmed adequate examiner calibration before measurement of the study specimens. During evaluation of the experimental specimens, the examiner remained blinded to the restorative material group to minimize measurement bias.

To minimize measurement bias, all specimens were coded before SEM evaluation using unique alphanumeric identification codes that did not disclose the restorative material or experimental group. Coding was performed by an investigator who was not involved in SEM imaging, marginal-gap measurement, or statistical analysis. The code-allocation key was retained separately and was not accessible to the examiner during measurement. Thus, the examiner performing the SEM-based marginal-gap measurements was blinded to the restorative material and group allocation of each specimen. The codes were decoded only after completion of all pre- and post-radiation measurements and finalization of the measurement dataset for statistical analysis.

The collected data were analyzed using IBM SPSS Statistics for Windows (version 25.0; IBM Corp., Armonk, NY). Data normality was assessed using the Shapiro-Wilk test. Descriptive statistics are expressed as mean, standard deviation, and 95% confidence intervals. Intergroup comparisons before and after radiation exposure were performed using one-way analysis of variance (ANOVA), followed by Tukey's honest significant difference (HSD) post-hoc test for pairwise comparisons. Intragroup comparisons between pre- and post-radiation measurements were performed using paired t-tests. The effect sizes were calculated using eta-squared (η²) for ANOVA and Cohen's d for paired comparisons. Statistical significance was set at p < 0.05.

## Results

Descriptive statistics and intergroup comparisons of the mean marginal gap before radiation exposure are presented in Table 2. One-way ANOVA demonstrated a statistically significant difference among the four restorative materials (F = 57.10, p < 0.001), with a large effect size (η² = 0.708). Among the tested materials, ormocer exhibited the lowest mean marginal gap, whereas giomer exhibited the highest mean marginal gap.

**Table 2:** Comparison of mean marginal gap (µm) among restorative materials before radiation exposure. Data are presented as mean ± standard deviation (SD) and 95% confidence interval (CI). Intergroup comparison was performed using one-way analysis of variance (ANOVA). Effect size was expressed as eta-squared (η²). * p < 0.05 was considered statistically significant.

Group	Restorative material	n	Mean ± SD	95% CI of mean	F value	p-value	Effect size (η²)
Group I	Nanocomposite	9	5.16 ± 0.93	4.96-5.36	57.10	<0.001*	0.708
Group II	Giomer	9	6.30 ± 0.42	6.00-6.60			
Group III	Ormocer	9	4.27 ± 0.32	4.02-4.52			
Group IV	SureFil SDR Flow Plus	9	6.24 ± 0.40	5.93-6.54			

The pairwise comparison of restorative materials before radiation exposure using Tukey's HSD post-hoc test is shown in Table 3. Statistically significant differences were observed between most pairwise comparisons (p < 0.05). However, no statistically significant difference was found between the giomer and SureFil SDR Flow Plus groups (P = 0.996).

**Table 3:** Pairwise comparison of mean marginal gap among restorative materials before radiation exposure. Data are presented as mean differences with corresponding test statistics (t) and p-values. Pairwise comparisons were performed using Tukey's honestly significant difference (HSD) post-hoc test following one-way ANOVA. * p < 0.05 was considered statistically significant.

Pairwise comparison	Mean difference (µm)	Test value (t)	p-value
Nanocomposite vs. Giomer	−1.14	−4.24	0.001*
Nanocomposite vs. Ormocer	0.89	3.31	0.012*
Nanocomposite vs. SureFil SDR Flow Plus	−1.08	−4.01	0.002*
Giomer vs. Ormocer	2.03	7.54	<0.001*
Giomer vs. SureFil SDR Flow Plus	0.06	0.22	0.996
Ormocer vs. SureFil SDR Flow Plus	−1.97	−7.32	<0.001*

An intergroup comparison of the mean marginal gaps after radiation exposure is shown in Table 4. One-way ANOVA demonstrated a statistically significant difference among the four restorative materials (F = 108.18, p < 0.001), with a large effect size (η² = 0.798). Ormocer demonstrated the lowest mean marginal gap following radiation exposure, whereas SureFil SDR Flow Plus exhibited the highest mean marginal gap.

**Table 4:** Comparison of mean marginal gap (µm) among restorative materials after radiation exposure. Data are presented as mean ± standard deviation (SD) and 95% confidence interval (CI). Intergroup comparison was performed using one-way analysis of variance (ANOVA). Effect size was expressed as eta-squared (η²). * p < 0.05 was considered statistically significant.

Group	Restorative material	n	Mean ± SD	95% CI of mean	F value	p-value	Effect size (η²)
Group I	Nanocomposite	9	6.22 ± 0.78	5.62-6.82	108.18	<0.001*	0.798
Group II	Giomer	9	6.96 ±0.32	6.71-7.21			
Group III	Ormocer	9	4.86 ± 0.30	4.63-5.09			
Group IV	SureFil SDR Flow Plus	9	7.08 ± 0.30	6.85-7.31			

A post-hoc pairwise comparison after radiation exposure is presented in Table 5. Significant differences were observed between most restorative materials (p < 0.05). Similar to the findings before radiation exposure, no statistically significant difference was observed between the giomer and SureFil SDR Flow Plus groups (p = 0.949).

**Table 5:** Pairwise comparison of mean marginal gap among restorative materials after radiation exposure. Data are presented as mean differences with corresponding test statistics (t) and p-values. Pairwise comparisons were performed using Tukey's honestly significant difference (HSD) post-hoc test following one-way ANOVA. * p < 0.05 was considered statistically significant.

Pairwise comparison	Mean difference (µm)	Test value (t)	p-value
Nanocomposite vs. Giomer	−0.74	−3.33	0.011*
Nanocomposite vs. Ormocer	1.36	6.11	<0.001*
Nanocomposite vs. SureFil SDR Flow Plus	−0.86	−3.87	0.003*
Giomer vs. Ormocer	2.10	9.44	<0.001*
Giomer vs. SureFil SDR Flow Plus	−0.12	−0.54	0.949
Ormocer vs. SureFil SDR Flow Plus	−2.22	−9.98	<0.001*

Intragroup comparison of the marginal gap values before and after radiation exposure is shown in Table 6. A statistically significant increase in the marginal gap was observed following radiation exposure for all four restorative materials (p < 0.05). The largest effect size was observed in the SureFil SDR Flow Plus group (Cohen's d = 2.38), indicating the greatest change in marginal adaptation after radiation exposure.

**Table 6:** Intragroup comparison of mean marginal gap before and after radiation exposure. Data are presented as mean ± standard deviation (SD). Mean difference was calculated as after radiation minus before radiation. Intragroup comparisons were performed using the paired t-test. Cohen's d was used to estimate effect size and interpreted as small (0.20), moderate (0.50), and large (≥0.80). * p < 0.05 was considered statistically significant.

Group	Restorative material	Mean difference (µm)	Test value (t)	p-value	Effect size (Cohen's d)
Group I	Nanocomposite	1.06	2.62	0.019*	1.24
Group II	Giomer	0.66	3.75	0.002*	1.77
Group III	Ormocer	0.59	4.04	<0.001*	1.90
Group IV	SureFil SDR Flow Plus	0.84	5.04	<0.001*	2.38

## Discussion

The present in vitro study evaluated the effect of therapeutic radiation on the marginal adaptation of four resin-based restorative materials: nanofilled composite resin, giomer, ormocer, and bulk-fill flowable composite. Marginal adaptation is a critical determinant of the clinical longevity of restorations because an increase in the marginal gap facilitates bacterial microleakage, postoperative sensitivity, marginal discoloration, recurrent caries, and ultimately, restoration failure [4-6]. The findings of the present study demonstrate that radiation exposure adversely affects the marginal adaptation of all restorative materials. However, the magnitude of deterioration varies according to the restorative material used.

Before radiation exposure, statistically significant differences in marginal gaps were observed among the four restorative materials. Ormocer exhibited the lowest marginal gap, indicating superior marginal adaptation, followed by nanocomposites, whereas giomer demonstrated the highest marginal gap. This finding is in agreement with the study by Yadav et al. [11]. Bollu et al. [12] reported significantly higher microleakage with giomer than with nano-ionomer and resin-modified glass ionomer cement, suggesting comparatively poorer marginal adaptation. Following radiation exposure, all restorative materials showed a significant increase in the marginal gap, confirming that therapeutic radiation negatively influences the integrity of the tooth-restoration interface [6]. Despite this increase, ormocers continued to exhibit the lowest marginal gap values, suggesting a greater resistance to radiation-induced degradation.

The superior performance of the ormocer may be attributed to its organically modified ceramic matrix, which contains an inorganic-organic hybrid network with lower polymerization shrinkage and reduced polymerization stress compared with conventional dimethacrylate-based composites [13]. The highly cross-linked three-dimensional structure of the monomer minimizes volumetric contraction during polymerization, resulting in improved adaptation to the cavity walls. Furthermore, its lower water sorption and enhanced dimensional stability may have contributed to the better preservation of the adhesive interface, even after radiation exposure. Monsarrat et al. [14] concluded that first-generation ormocers did not exhibit a significant clinical advantage over conventional composites but suggested that newer pure ormocer formulations may provide improved stability and resistance. The favorable marginal adaptation observed with ormocer in the present study may reflect these advances in ormocer technology.

The nanofilled composite demonstrated intermediate marginal adaptation before and after radiation. The presence of nanosized filler particles improves filler loading, polishability, and mechanical properties; however, the conventional methacrylate resin matrix remains susceptible to polymer degradation and hydrolytic changes following exposure to ionizing radiation [7]. These alterations may weaken the adhesive interface and contribute to the increased marginal gap formation after irradiation. Lawson and Burgess [15] reported that an appropriate nanofiller concentration improves the mechanical properties of nanofilled composites, including their hardness and flexural strength. These characteristics may partially explain the favorable marginal adaptation of the nanofilled composites observed in the present study. Sharma et al. [16] reported a significantly greater microleakage with nanocomposite restorations than with microhybrid composites. This finding differs from that of the present study and may be attributed to differences in the restorative materials, curing units, experimental design, and the inclusion of radiation exposure in the present investigation.

The giomers exhibited comparatively higher marginal gap values in both the pre-radiation and post-radiation evaluations. Although giomers possess surface pre-reacted glass ionomer (S-PRG) fillers capable of fluoride release and recharge, their relatively higher water sorption and resin matrix characteristics may adversely influence dimensional stability [17]. Radiation-induced alterations in the resin matrix and filler-matrix interface may further compromise the marginal integrity, explaining the greater marginal gaps observed in the present study. Similarly, SureFil SDR Flow Plus demonstrated a significant increase in the marginal gap following radiation exposure, and showed the greatest overall change among the evaluated materials. Although bulk-fill flowable composites are designed to reduce polymerization stress using stress-relieving monomer technology, their lower filler loading and relatively higher resin content may render them more susceptible to radiation-induced degradation and dimensional alterations, resulting in increased marginal gap formation [7,18].

The findings of the present study are consistent with those reported by Shono and Alkhudhairy [19], who demonstrated significant differences in marginal adaptation among resin-based restorative materials and reported superior marginal adaptation with ormocer-based composites compared to conventional resin composites. Their study attributed these findings to the reduced polymerization shrinkage and improved dimensional stability of ormocer materials, which agrees with the observations of the present study. Previous investigations evaluating the influence of radiotherapy on restorative materials have also reported deterioration in marginal integrity following irradiation. Experimental studies have shown that therapeutic radiation alters the physical and chemical properties of resin matrices, reduces bond strength, and promotes degradation of the adhesive interface, thereby increasing marginal discrepancies [5,6]. The present findings are in accordance with these reports and further demonstrate that the extent of radiation-induced deterioration is material dependent.

The findings of the present study demonstrated that therapeutic radiation significantly increased the marginal gap of all restorative materials, although the magnitude of change varied according to material composition. These observations are consistent with the systematic review by Troconis et al. [20], who concluded that while the mechanical properties of composite resins are generally maintained following head and neck radiotherapy, the bond strength of adhesive systems tends to decrease when bonding is performed after radiation exposure, potentially compromising the tooth-restoration interface. Similarly, Ugurlu et al. [21] reported that the effects of ionizing radiation are material-dependent and influenced by the chemical composition of restorative materials, with significant alterations observed in surface characteristics and fluoride release following irradiation. Furthermore, de Amorim et al. [22] demonstrated that exposure to ionizing radiation altered the surface micromorphology of all tested restorative materials, although resin composites exhibited greater resistance to radiation-induced changes than did glass ionomer-based materials. These findings support the present study, in which all restorative materials exhibited increased marginal gaps after radiation exposure, but ormocer demonstrated superior marginal adaptation, suggesting that differences in resin matrix composition and material characteristics play an important role in determining the resistance to radiation-induced degradation.

Clinical implications

From a clinical perspective, these findings may assist clinicians in selecting restorative materials for patients undergoing head and neck radiotherapy. Since radiation exposure may compromise the tooth-restoration interface and increase marginal discrepancies, materials capable of maintaining greater dimensional and marginal stability may be preferable in this patient population. Among the materials evaluated, the ormocer-based composite demonstrated the most favorable marginal adaptation both before and after irradiation, suggesting that it may be considered a suitable restorative option when direct resin-based restorations are required in teeth expected to be exposed to therapeutic radiation. Nevertheless, material selection in daily clinical practice should also consider other relevant factors, including the location and extent of the lesion, remaining tooth structure, adhesive conditions, oral hygiene, salivary function, caries risk, and anticipated radiation exposure. As the present findings were obtained under in vitro conditions, they should support rather than independently determine clinical material selection, and confirmation through long-term clinical studies is warranted.

Limitations

The present study had certain limitations. As an in vitro investigation, factors such as saliva, thermal fluctuations, masticatory loading, pH changes, oral microbiota, and long-term aging could not be reproduced. Only one adhesive system and one representative material from each restorative category were evaluated. Furthermore, the findings are specific to standardized class V cavities with occlusal margins in enamel and cervical margins in dentin and should not be generalized to other cavity configurations. Longitudinal sectioning before irradiation, although allowing paired measurements, may have altered specimen integrity and the response of the tooth-restoration interface to radiation. Additionally, a formal material × irradiation interaction was not assessed using a mixed-design model. Therefore, the findings should be interpreted cautiously. Future studies using non-destructive assessment methods, different cavity designs, thermocycling, mechanical loading, long-term aging, and clinical evaluation are recommended.

## Conclusions

Within the limitations of this in vitro study, therapeutic radiation significantly increased the marginal gap within all evaluated restorative material groups. Under the standardized class V cavity design and adhesive protocol using Single Bond Universal Adhesive, ormocer demonstrated the lowest marginal gap values both before and after irradiation. However, as a formal material and irradiation interaction was not evaluated, these findings should not be interpreted as establishing differential resistance to radiation among the restorative materials. The results are specific to the materials, adhesive system, and experimental conditions evaluated and should be interpreted cautiously when extrapolated to clinical practice.
